# Exploring nocturnal soundscapes in an Australian open forest system using acoustic indices

**DOI:** 10.1371/journal.pone.0348624

**Published:** 2026-05-15

**Authors:** Felipe do Carmo Jorge, Gabriel Politzer Couto, Berndt J. van Rensburg

**Affiliations:** 1 School of the Environment, Centre for Biodiversity and Conservation Science, University of Queensland, Brisbane, Queensland, Australia; 2 Department of Zoology, University of Johannesburg, Johannesburg, South Africa; Shenyang Jianzhu University, CHINA

## Abstract

Nocturnal environments and their ecology remain noticeably under-represented in the scientific literature, despite their ecological importance, largely due to the practical challenges of sampling nighttime ecology. Passive acoustic monitoring provides a cost-effective, scalable tool for extracting ecological insights from continuous recordings, using acoustic indices. This study assessed whether a suite of acoustic indices can capture the diversity of nighttime calls (i.e., sonotype richness used here as a proxy for biodiversity) in a terrestrial ecosystem. Our main findings supported an interaction effect of indices (acoustic activity (ACT), acoustic evenness (AEI), spectral entropy (Hf), soundscape saturation (Sm)) correlating best with variation in sonotype richness. This result was further supported by habitat-dissimilarity analyses based on manually tagged insect sonotype richness compared to index values showing significant similarities. Although the methodological framework used in this study is adaptable to other environments, index performance is unlikely to be directly transferable due to differences in vocal assemblages and background noise conditions. Overall, the study provides a useful procedure to track patterns in nighttime ecology and support the evaluation of the state of nighttime ecology.

## Introduction

The need to better understand the ecology of the nighttime has long been highlighted by ecologists (see, e.g., [1]) and recent efforts have been made to highlight the importance of re-establishing this research topic (see, e.g., [2]). An important factor that underpins this research topic relates to the high biodiversity value associated with the nighttime. Considering nighttime ecology in research programs and conservation decision making is therefore essential knowing the crucial role that invertebrates play in ecosystems [3] (see the recent study by [4] indicating that, at a global scale, insect activity and abundance are significantly higher at night than in daytime). Nocturnal environments are therefore key to help sustain food webs, pollination networks, and play a crucial role in ecosystem functioning and resilience [5,6].

Despite their ecological significance, studies focusing on nocturnal environments and their ecology remain noticeably under-represented within the scientific literature when compared with studies focusing on the ecology of the daytime [2]. Nocturnal terrestrial animals consist of approximately 30% of all known vertebrate species and more than 60% of invertebrates, yet they are often neglected from assessment due to experimental challenges [7]. Ways to narrow this knowledge gap is becoming increasingly urgent as invertebrate diversity, which constitutes the majority of nocturnal species, is declining globally at an unprecedented rate [8–10]. Furthermore, nocturnal biodiversity is often overlooked in conservation decision-making due to the many practical challenges in studying organisms at night [11]. Addressing this gap and testing new and improved technologies to monitor how nighttime organisms respond to management actions are all part of what is needed to overcome some of the challenges that contributes towards the under-representation of nighttime ecology within the scientific literature.

Within the nocturnal soundscape, biophony comprises numerous vocal taxa, making it ecologically valuable yet challenging for species identification and assessing their conservation status. Some terrestrial taxa, such as insects, exhibit high diversity but lack sufficient expert taxonomists, hindering routine monitoring efforts [12]. This challenge highlights the need for alternative approaches like taxonomic sufficiency [13], which seeks the minimal level of taxonomic resolution necessary to tackle ecological assessments while accounting for practical and logistical constraints. For example, the morphospecies approach has been widely used as a surrogate for richness in groups with limited species-level taxonomic knowledge (see, e.g., [12] for ants). However, prior knowledge of local species and their ecology is crucial for selecting suitable biodiversity surrogates when species-level identification is not feasible [13,14], and its effectiveness varies across taxonomic groups [15]. In bioacoustics, the scaling of passive acoustic monitoring [16], from which species calls can be used to assess the composition of an assemblage has been demonstrated across a range of vocal taxa (see, e.g., [17]; but also see [18] for limitations). Such scaling is an example of a surrogate approach within bioacoustics as a proxy for biodiversity, and this has been shown to be particularly suitable for nocturnal vocal species [4,19].

Soundscapes provide an important source of information about ecosystems, offering valuable insights into nocturnal ecology and biodiversity that are often overlooked. In this context, despite care to be devoted to their interpretation and the tools that AI is introducing in species recognition, acoustic indices calculated from soundscape recordings can be used to estimate species diversity by capturing the acoustic footprint left by vocal signals – a computational process that quantifies signal amplitude across both frequency and time domains [20,21]. In theory, greater vocal species diversity leads to a soundscape that has spectrograms characterised with more densely filled acoustic energy. This approach is grounded in the theory of acoustic niche partitioning, which proposes that coexisting vocal species in preserved habitats share the acoustic space by avoiding signalling overlap as result of ecological processes such as evolution, natural selection, and time [22–24]. Therefore, a diverse array of vocal signal types, or “sonotypes,” is expected to be detectable in the outputs of acoustic indices. These indices have been widely used as proxies for biodiversity in terrestrial ecosystems, as well for assessing ecological condition [24,25], although their accuracy in fully capturing some environmental parameters and diversity facets remains a subject of ongoing debate [18].

Studies applying terrestrial PAM to characterise nocturnal soundscapes, with a particular focus on insect assemblages, remain limited [26], whereas PAM is more commonly designed around daytime bird surveys, nocturnal bat monitoring, or continuous 24-hour recording cycles [27,28]. From recordings, data extraction typically follows three main approaches: manual annotation of vocalisations [29], machine-learning species classifiers [30], and the use of acoustic indices as proxies for diversity [17,31–33]. For example, indices have been used to track variation of insect activity in 24-hour PAM recordings from Brazilian savannas [17] and Amazon forest [32,33]. Although these studies highlighted the value of acoustic indices as useful tools for tracking nighttime ecology by quantifying patterns in soundscapes, they also suggested the need to interpret the utility of these indices with caution (e.g., loud cicada choruses can bias index-based inferences by masking other biophony; see [34]), and that applying them across different ecosystems presents several challenges and limitations requiring further research.

For this study, we use sonotype richness as a measure of terrestrial nighttime biodiversity [17,32,33]. The broader aim of this study is to assess the value of acoustic indices to reflect variation in nighttime sonotype richness. To address this aim, we first examined, both as a main effect (i.e., in isolation) and as an interaction effect, the extent to which acoustic indices correlate with sonotype richness (response variable). We further investigated this aim by comparing annotated sonotype richness values with acoustic index values using a distance matrix approach. We argue that if the distances between index values closely reflect that of the annotated richness values, then that further validate the use of indices to describe sonotype richness (i.e., biodiversity). This study therefore aims to further our knowledge within the field of bioacoustics by identifying the value of acoustic indices for assessing systems that are often poorly studied such as nocturnal environments and providing a practical application of using soundscapes for assessing biodiversity. Limitations and best practices of these indices for future nighttime monitoring are also considered.

## Materials and methods

### Study area

**Fig 1:**
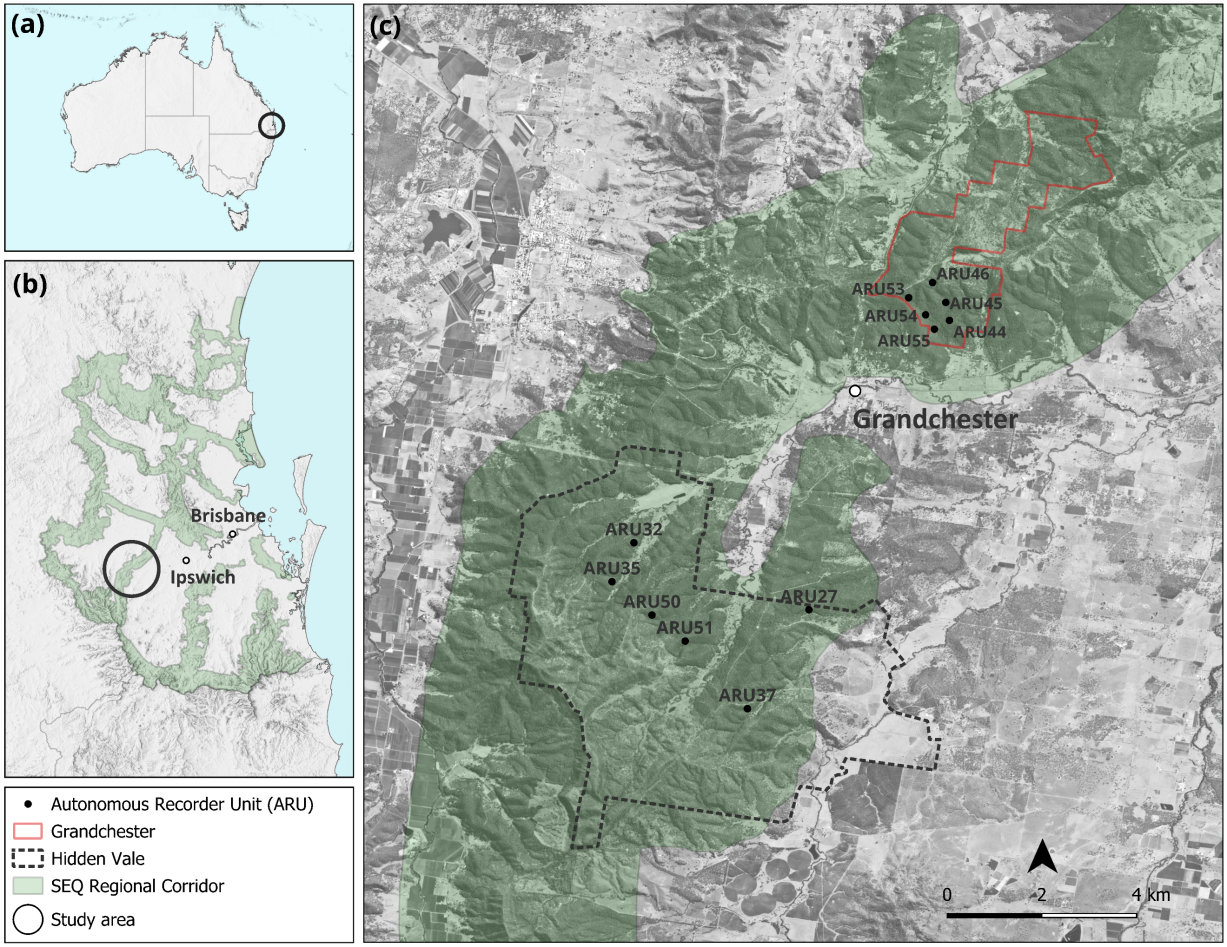
Recording sites monitored during summer Jan-Feb 2024 at Hidden Vale Research Station (hereafter, Hidden Vale) and Mount Grandchester Conservation Estate (hereafter, Grandchester). (a) location of the study area within Southeast Queensland, Australia (circled); (b) Southeast Queensland (SEQ) Regional Corridor, with the Little Liverpool Range (circled) linking the Main Range National Park and the Great Eastern Ranges; (c) boundaries of the two study areas and locations of Autonomous Recording Units (ARUs). Republished from *Queensland LiDAR Data – Ipswich 2019 Project* under a CC BY license, with permission from the Department of Natural Resources and Mines, Manufacturing and Regional and Rural Development, original copyright *The State of Queensland 2025*.

The acoustic data were collected in twelve survey sites within southeast Queensland, Australia spanning two reserves – six sites in the Hidden Vale Research Station located at 27°42’23“ S, 152°25’1” E (hereafter, Hidden Vale), and six in the Mount Grandchester Conservation Estate located at 27°38’44” S, 152°28’42” E (hereafter, Grandchester) (Fig 1).

This study involved passive acoustic monitoring of forest soundscapes using 24-hour recording schedules and did not involve the capture, handling, or direct observation of animals or human participants. Field research was conducted under Permit No. 18693, issued by Ipswich City Council pursuant to local laws, authorizing scientific research (recording of bird calls) at Mount Grandchester Conservation Estate on approved dates between June 2023 and May 2024. Additional access permissions were granted by the Hidden Vale Research Station (University of Queensland). No further ethical approval was required.

The local regional ecosystem for both study areas is RE 12.9–10.2, characterized by open forest structure dominated by *Corymbia citriodora* (Lemon-scented Gum) and *Eucalyptus crebra* (Narrow-leaved Ironbark), typically found on sedimentary rock substrates. The study area is situated on hilly terrain, consisting of a mosaic of preserved patches, grazing lands, and logged areas [35]. Although this regional ecosystem is classified under the Vegetation Management Act as “Least concern,” indicating it is not currently at significant risk of decline [36], remnant patches within the two selected study areas form part of the Little Liverpool Range, an ecological corridor connecting the Main Ranges National Park and the D’Aguilar National Park (the Great Eastern Ranges) [37,38] (Fig 1). From a conservation perspective, these two study areas, and the broad ecosystem they represent, are therefore important (a) geographically within the context of conserving ecological corridors, and (b) for land managers knowing that habitat restoration, and the way in which biodiversity responds to these management actions, is a high priority within both these study areas [37,38].

### Recording scheme

We used autonomous recorders units (ARU) manufactured by Frontier Labs (BAR-LT with standard omnidirectional microphone for which a flat response of +-2dB from 80 Hz to 20kHz was assumed). Consistency in microphone gain was assessed by coupling a portable acoustic calibrator (TekcoPlus Ltd., model SLM-111) to each microphone and verifying its sensitivity to a tone (94 dB SPL at 1 kHz); sensitivities were consistent across microphones (mean ± SD = −26.86 ± 0.45 dB; see full calibration table in the DOI repository). Twelve recorders (one in each survey site) were deployed with a minimum distance of 400 m between sites. The devices were programmed to record 30 days (January-February 2024 representing the summer season which is the peak nocturnal animal vocalization season for the study area) continuously 24 hours at 44.1kHz (mono, 16 bits WAV, 40 dB gain). As the sites were situated within two forest reserves, they were all free from artificial light exposure. However, anthropophony from the highway and train rail crossing between the two reserves was frequently detected in recordings, primarily occupying the very low-frequency bands between 0–300 Hz (see S3 Fig for the mean power spectrum values per site).

### Data analyses

Five non-consecutive nights were selected from each of the twelve sites (January 8th, 17th, 21st, 26th, and February 2nd of 2024). Nights were selected based on manual inspections of the recording quality, aiming to minimize the effects of geophony (nights with calm weather conditions with minimum wind). This approach provided broad temporal coverage of summer soundscapes under consistent weather conditions, with the same five nights sampled at all sites to minimize environmental variation in vocal activity and to enable comparable sampling of the local vocal community across sites.

From each night’s recording, a 2.5-hour window per site was used for data extraction starting approximately one hour after sunset (19:30–22:00). This timing minimized contributions from dusk-active species (some of which are also diurnally active) while targeting the onset of nocturnal Orthopteran activity (crickets, katydids, grasshoppers, and locusts) during the reproductive (summer) season. Each 2.5-hour window was segmented into 1-minute clips at 10-minute intervals, resulting in 15 1-minute clips per night, from which one 1-minute clip was randomly selected for analysis. To assess whether this sampling approach was appropriate, we examined the empirical distribution of full-spectrum power among all candidate audio clips. For each clip, mean power spectral density (PSD) was calculated within consecutive 1-kHz bands spanning 0–20 kHz, summed in linear units across bands, and then converted to relative dB. The resulting distribution was unimodal and broadly continuous across the candidate pool (S1 Fig), and spectral structure remained broadly consistent across the 15 time points within the 2.5-hour sampling window (S2 Fig). Together, these patterns supported the use of one randomly selected 1-minute clip from each 2.5-hour window. With six sites per study area (and five nights per site), this resulted in 60 1-minute sample recordings (30 per study area) providing a comprehensive temporal and spatial coverage of the summer soundscape within the broader study region (audio files are available in DOI repository).

Following that of [17,23], sonotypes were manually annotated by listening to recordings and examining spectrograms within each 1-minute sample. Several public web databases, such as [39–41] were consulted for reference calls to assist with the identification and assignment of sonotypes to taxonomic groups within the biophony (sonotype description in S1 Table). Four taxon groups were identified: frogs, nocturnal birds, and mammals below 4,000 Hz (with only a bat sonotype annotated above 10,000 kHz); and insects (predominantly Orthopterans; i.e., crickets, grasshoppers, katydids, and locusts) between 1,400 and 20,000 Hz (with the majority of these insect calls between 3,500–10,000 Hz) (see S1 Table and sonotype samples in the DOI repository). A presence–absence sonotype matrix was compiled to assess richness (rows = site × night recordings; columns = sonotypes; 1 = detected, 0 = absent). All soundscapes present across the 60 1-minute samples were annotated and assigned to the four taxon groups (insects, birds, frogs, mammals) and to “other” (noise from anthropophony or geophony; presence–absence sonotype matrix available in DOI repository).

The same 60 1-minute audio samples as described above were used to calculate four acoustic indices that quantify temporal and spectral structure: acoustic activity (ACT) [21], acoustic evenness (AEI) [42], spectral entropy (Hf) [43], and soundscape saturation (Sm) [44]. Together, AEI, Hf, and Sm characterise the distribution and spread of acoustic energy across frequency bands, while ACT provides a complementary measure of overall acoustic activity over time. All these indices are expected to reflect patterns associated with vocal diversity and its use of acoustic space. These were computed using: (1) the unfiltered 1-minute files (hereafter, raw-1minute) and (2) the raw files after applying a high-pass (low-cut) filter (hereafter, filtered 1-minute files) at 300 Hz using the seewave bwfilter function (i.e., retaining 300–20,000 Hz) to attenuate low-frequency anthropogenic noise, in R software [45–48]. In the raw recordings, dominant low-frequency energy elevates background noise levels and reduces the effective signal-to-noise ratio (SNR) of low-amplitude biophonic components, which can mask signals and bias threshold-based metrics (particularly Sm). This masking is evident in the power spectrum, where power spectral density (PSD) above ~10 kHz (including insect components) occurs at substantially lower levels than the low-frequency anthropogenic band (S3 Fig). After high-pass filtering, reduced low-frequency noise increases effective SNR across the retained frequency range (S4 Fig). ACT represents the proportion of time bins where the amplitude envelope exceeds a fixed threshold of 5 dB above the 5th-percentile envelope, using msmooth = c(200, 0) for envelop smoothing. High ACT values indicate greater acoustic activity. AEI divides the spectrogram into 1000 Hz frequency bands and, for each band, computes the proportion of samples exceeding a −45 dBFS threshold, then calculated as the Gini coefficient of those band-wise proportions (values range from 0 to 1, with 0 indicating equal energy distribution). Hf is an adaptation of the Shannon index, where frequency bands are treated as “species” and their relative amplitudes are normalized analogous to species abundances (the index ranges from 0 to 1, reflecting the equitability of energy distribution across bands). Sm calculates the percentage of frequency bins whose SNR exceeds a predefined threshold. Accordingly, Sm thresholds were set to 6.3 dB for raw 1-minute files and 3.8 dB for filtered files. Thresholds were selected empirically by screening values from 3.0 to 8.0 dB in 0.1 dB increments and choosing the lowest threshold that (i) avoided a ceiling effect (Sm approaching 100%) and (ii) maximised model performance, quantified as the highest GLM R^2^ for models predicting biophony and insect richness. Indices were calculated in R [45] using tuneR [46], seewave [47], and soundecology [48]. For Hf and Sm, we used a frequency bin width of ~90 Hz and a temporal resolution of ≈11 ms (wl = 484, ovlp = 90% at sample rate of 44.1kHz).

(i) **The value of acoustic indices to reflect variation in nighttime sonotype richness**

For this analysis, acoustic indices were averaged across the five sampling nights at each site, and examined, both as a main effect (i.e., in isolation) and as an interaction effect (i.e., equal-weight sums of these standardised indices) [21]) and paired with that site’s accumulated sonotype richness. Although it reduces the sample size from 60 1-minute clips (5 nights per site x 12 sites) to 12 1-minute clip (i.e., one clip per site; and each site clip represents the mean across 5 nights), the means were found to provide stronger models when examining the relationship between acoustic indices and sonotype counts during preliminary tests (see S2 and S3 Tables). Site-level sonotype richness was defined as the number of distinct sonotypes manually annotated at least once across the five nights (accumulated presence–absence). We used this metric instead of mean sonotype richness because it provides more robust and representative site-level estimate for comparison with acoustic indices (see S4 Table). The sonotype counts were grouped into three response variables for analysis: (a) Biophony richness (the sum of annotated sonotypes from insects, frogs, birds and mammals), (b) Insect richness only, assessed separately due to their dominance in summer nocturnal soundscapes, ecological importance, and limited knowledge [13,49], and (c) a multi-taxon group combining frogs, nocturnal birds, and mammals, which typically vocalised below ~3.5 kHz.

For the interaction-effect analysis, all acoustic indices (ACT, AEI, Hf, Sm) were first standardised within the dataset using z-scores, $z(x)=(x-\bar{x})/s_{x}$, where $\bar{x}$ and are the mean and standard deviation across sites, respectively. Equal-weight composite indices were then formed by summing these standardised components (e.g., Hf + Sm; AEI + Hf + Sm; ACT + AEI + Hf + Sm; ACT + Hf + Sm). Each composite was re-standardised (z-scored again) to ensure a mean of 0 and unit variance, thereby placing composites with different numbers of components on a common scale. This two-stage procedure ensures unit invariance, equal component weighting, and comparability of effect sizes across single indices and composites. Pairwise richness models used these z-scored indices or composites as the sole continuous predictor, with Reserve (i.e., study area) included as a fixed factor. Slopes were thus interpreted as the expected change in sonotype richness for a one–standard-deviation increase in the index or composite, holding Reserve constant.

We fitted generalized linear models (log link) in glmmTMB to relate site-level sonotype richness (Biophony, insects and frogs/birds/mammals) to acoustic indices. For each response-predictor combination, we evaluated alternative count-data distributions: Poisson, negative binomial (NB1 and NB2), generalized Poisson, and Conway–Maxwell–Poisson. Models were selected primarily by the lower AIC. When candidate models were within ΔAIC < 2, we broke ties by preferring the model whose Pearson dispersion (χ²/df) was closest to 1.

Predictors included ACT, AEI, Hf, and Sm, as well as their composite interactions: (Hf + Sm), (ACT + Hf + Sm), (AEI + Hf + Sm), and (ACT + AEI + Hf + Sm). For each selected model we report the significance (p-value), Efron’s R² and AIC (Akaike Information Criterion). This procedure was conducted on the raw 1-minute audio files and on filtered 1-minute files (300–20,000 Hz).

(ii) **Comparing raw annotated sonotype richness values with acoustic index values**

Dissimilarity between sonotype richness and index values was quantified (using a distance matrix approach) for the best predictor model identified from our first set of analysis (i.e., assessing the value of acoustic indices to reflect variation in nighttime sonotype richness). For the annotated sonotype richness, site-level presence–absence matrices were analysed with binary Jaccard distance (vegan::vegdist, method = “jaccard”, binary = TRUE). For acoustic indices, site means were used, and dissimilarity was computed with the Ružička distance (quantitative Jaccard; vegan::vegdist, method = “ruzicka”). For interaction indices, site-mean ACT, AEI, Hf, and Sm were range-scaled to [0,1] (with ACT inverted to align directionality) prior to computing the Ružička distance.

Non-metric Multidimensional Scaling (NMDS) was applied to each distance matrix to visualise the sites dissimilarity. Site scores were grouped by study area, and normal ellipses were drawn to summarise within-reserve dispersion (reported as the corresponding coverage level). Complementing this approach, the mean dissimilarity between sonotype richness and acoustic indices value for all cross-study areas site pairs, and the mean within-study areas dissimilarity were calculated. Statistical evaluation involved Permutational Multivariate Analysis of Variance (PERMANOVA) with 9,999 permutations to test for significant differences in sonotype richness and soundscape structure between study areas. Significance was quantified with PERMANOVA p-values and effect magnitude with PERMANOVA R². For within-study areas evaluation, a Permutational Test of Multivariate Dispersion (PERMDISP) was conducted using the same Jaccard distance matrices, with PERMDISP p-value indicating whether the dispersion of sites around their study area centroids differed significantly between study areas. This comprehensive framework provides a thorough understanding of soundscape structure of each study area and the practical application of acoustic indices as proxies for vocal species richness.

## Results

Acoustic index results suggest that both study areas share similar nocturnal vocal diversity and soundscape structure patterns (Table 1), which was captured by the interaction of indices. The sum of z-scored acoustic activity (ACT), acoustic evenness (AEI), spectral entropy (Hf), soundscape saturation (Sm) produced the most significant predictive model to predict insect sonotype richness in the filtered dataset and AEI, Hf and Sm for Biophony from the raw 1-min audio files. Importantly, these metrics converged on a consistent ecological interpretation of community dissimilarity within and between study areas: both insect sonotype richness and indices interaction (ACT, AEI, Hf, Sm) indicated that between-reserve dissimilarity exceeded within-reserve dissimilarity, and Grandchester was slightly more heterogeneous than Hidden Vale. Insect composition showed Jaccard dissimilarities of 0.55 between reserves and 0.54–0.50 within reserves, while the indices composite showed lower absolute dissimilarities (0.38 between; 0.40–0.35 within) but mirrored the same spatial ordering.

**Table 1:** Summer season summary of nocturnal sonotype richness and corresponding acoustic indices for Grandchester and Hidden Vale reserves. Acoustic activity (ACT), acoustic evenness (AEI), spectral entropy (Hf), soundscape saturation (Sm). Bio = biophony (all vocal sonotypes); Ins = insects; Bir = birds; Fro = frogs; Mam = mammals; Filt = filtered audio files (0.3-20kHz); Raw = raw audio filles (0-20kHz).

Reserve	Bio	Ins	Bir	Fro	Mam	ACT		AEI		Hf		Sm	
						Filt	Raw	Filt	Raw	Filt	Raw	Filt	Raw
Grandchester	52	33	7	5	7	52	49	0.44	0.44	0.81	0.82	95	38
Hidden Vale	49	29	7	6	7	39	38	0.42	0.42	0.83	0.83	95	43

(i) **The value of acoustic indices to reflect variation in nighttime sonotype richness**

Generalized Linear Models indicated that the interaction effect between indices ACT, AEI, Hf and Sm explained most of the variation (smallest AICc scores and most significant) for insects from the filtered audios (p < 0.0001, R² = 0.77) and Biophony sonotype richness from the raw audios (p < 0.001, R² = 0.59; Table 2). Considering the main effects of the indices (i.e., in isolation), AEI significantly predicted Frog/Bird/Mammal richness (p < 0.02, R² = 0.44), and Hf significantly predicted biophony richness in both datasets (filtered and raw audio) (p < 0.01, R² ~ 0.36; Table 2). No index’s in isolation were found to significantly explain variation within insect richness (Table 2). In contrast, clip-level GLMMs showed weaker support overall: with Site fitted as a random intercept, only the full composite significantly predicted filtered frog/bird/mammal richness (p = 0.03, R² = 0.12; S2 Table), and when both Site and Night were included as random intercepts, no significant relationships remained (S3 Table). Models based on mean site-level richness were also weaker for representing insects, the biophony group most representative of the nocturnal soundscape in summer, than those based on accumulated site-level richness. Under mean richness, the insect predictive model from the filtered dataset remained significant (p = 0.003, R² = 0.43), as did the biophony model from the raw dataset (p = 0.052, R² = 0.26), while Frog/Bird/Mammal richness was significantly related to AEI (p = 0.005, R² = 0.57; S4 Table). Thus, the larger effect sizes observed for the insect and biophony predictive models supported the use of accumulated site-level richness in the main analysis.

**Table 2:** Generalized Linear Models of annotated sonotype richness versus acoustic indices calculated from nocturnal soundscapes across 12 sites, using predictor variables as main effects (ACT, AEI, Hf, Sm) and interaction effect (equal-weighted sum of z-scored indices), with response variables total biophony richness and insect richness. Indices are acoustic activity (ACT), acoustic evenness (AEI), spectral entropy (Hf) and soundscape saturation (Sm). Datasets: filtered audio files (0.3-20kHz) and raw audio filles (0-20kHz). Biophony = the sum of annotated sonotypes from insects, frogs, birds and mammals. Insect = the sum of annotated sonotypes, predominantly from crickets, grasshoppers, katydids, and locusts.

Dataset	Response	Index	p-value	R²	AICc
Filtered	Insects	z-scored (ACT, AEI, Hf, Sm)	0.000000001	0.77	54
Filtered	Frogs/Birds/Mammals	AEI	0.02	0.44	55
Filtered	Frogs/Birds/Mammals	z-scored (ACT, AEI, Hf, Sm)	0.02	0.42	55
Filtered	Biophony	z-scored (ACT, AEI, Hf, Sm)	0.003	0.45	59
Filtered	Biophony	z-scored (AEI, Hf, Sm)	0.004	0.42	59
Filtered	Biophony	Hf	0.01	0.37	60
Filtered	Biophony	z-scored (ACT, Hf, Sm)	0.01	0.35	61
Filtered	Biophony	z-scored (Hf, Sm)	0.02	0.31	61
Filtered	Insects	z-scored (AEI, Hf, Sm)	0.002	0.45	64
Raw_1 min	Biophony	z-scored (AEI, Hf, Sm)	0.00006	0.59	55
Raw_1 min	Frogs/Birds/Mammals	AEI	0.02	0.44	55
Raw_1 min	Biophony	z-scored (ACT, AEI, Hf, Sm)	0.002	0.45	59
Raw_1 min	Biophony	Hf	0.01	0.35	61
Raw_1 min	Biophony	z-scored (Hf, Sm)	0.03	0.3	61
Raw_1 min	Insects	z-scored (AEI, Hf, Sm)	0.03	0.29	67

(ii) **Comparing raw annotated sonotype richness values with acoustic index values**

**Fig 2:**
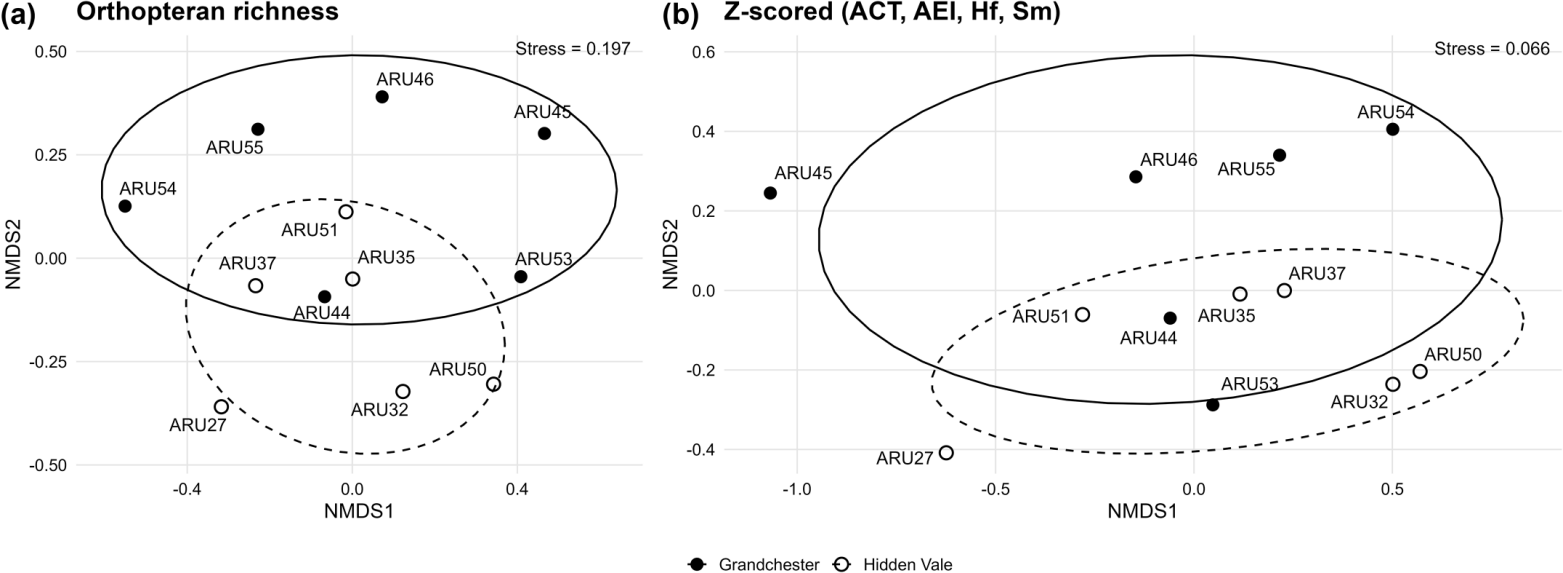
Non-metric multidimensional scaling of (a) Insect sonotype richness (Jaccard binary) and (b) indices interaction (acoustic activity (ACT), acoustic evenness (AEI), spectral entropy (Hf), soundscape saturation (Sm)) scaled composite index with Ružička distance) comparing Hidden Vale and Grandchester. Points are labelled by survey site and coloured by reserve; ellipses were drawn using the within-reserve mean dissimilarity distance. Stress values are reported for each ordination, with small values indicating closer similarity.

The parameters from the best predictive model in Table 2 were applied to the Non-metric multidimensional scaling ordinations: (a) Insects sonotype richness and (b) the interaction effect of indices ACT, AEI, Hf and Sm. Both ordinations show that Grandchester and Hidden Vale sites are arranged in highly similar configurations, highlighting that, as expected, the two study areas share similar sonotype richness. The ordination geometries are highly concordant, with recurring site neighbourhoods in the shared zone (e.g., ARU35, ARU37, ARU44, BAR51), indicating that the indices capture the same ecological pattern as the insect sonotype richness. Ellipses overlap broadly in both panels, consistent with the non-significant PERMANOVA and comparable within-study areas dissimilarities. Stress values (Insects = 0.197; indices = 0.066) indicate an acceptable two-dimensional fit, particularly robust for the indices (Fig 2).

Pairwise dissimilarity between study areas was higher for insect richness (55%) than for indices composite (38%), but both perspectives showed the same spatial ordering: between-reserve dissimilarity exceeded within-reserve dissimilarity, and Grandchester was slightly more heterogeneous than Hidden Vale (Insects: 0.54 vs 0.50; indices: 0.40 vs 0.35; Table 3). PERMANOVA provided no evidence of differences between study areas for either metric (Insects: p = 0.11; R² = 0.14; indices = p = 0.38, R² = 0.09) and PERMDISP indicated comparable dispersion between reserves (Insects: p = 0.28; indices: p = 0.73) (Table 3).

**Table 3:** Between and within similarity metrics of Hidden Vale and Grandchester from PERMANOVA and PERMDISP based on insect richness (sonotype count presence on five non-consecutive nights in summer season) and indices interaction (acoustic activity (ACT), acoustic evenness (AEI), spectral entropy (Hf), soundscape saturation (Sm)) calculated over the same audio clips. PERMANOVA R² and PERMANOVA p-value quantify centroid separation; PERMDISP p-value tests equality of multivariate dispersion, whereas non-significant indicates similar spread; Within Grandchester and Within Hidden Vale are mean pairwise distances among sites within each reserve, with lower values indicate more homogeneous.

Metric	Between dissimilarity	Within Grandchester	Within Hidden Vale	PERMANOVA p-value	PERMANOVA R²	PERMDISP p-value
Insect richness	0.55	0.54	0.5	0.11	0.14	0.28
Indices	0.38	0.40	0.35	0.38	0.09	0.73

## Discussion

Although the approach of applying acoustic indices to monitor patterns in soundscapes is not novel, it’s application focusing specifically on nighttime ecology remains largely understudied at a global scale and not attempted previously for an ecosystem within Australia. This study underscores the potential of using soundscape analysis with acoustic indices as surrogates for assessing nocturnal biodiversity focusing on sonotype richness within an open forest ecosystem in southeast Queensland. The main findings demonstrate that using an interaction of acoustic indices can be effective to assess nocturnal soundscapes in the summer season, which primarily reflect insect vocal diversity. The scalability and practicality of passive acoustic monitoring, combined with the high diversity of vocal taxa being active at night, emphasize the importance of incorporating ecoacoustic methods when considering, for example, animal responses to habitat management actions.

Considering main effects (i.e., the indices in isolation), AEI was the most significant predictor of frogs, nocturnal birds and mammals, followed by Hf for overall biophony (the sum of all sonotypes present including insect, frogs, birds, and mammals). ACT and Sm were not statistically significant for any assessed taxon in this dataset (see full results in S5 Table). This pattern was consistent for both the raw and the filtered recordings. For predicting insect sonotype richness only, main-effect models were not significant.

At least for our study, the best model performance emerged from an interaction (i.e., equal-weighted, z-scored sum) of all four indices (for both the filtered and the raw recordings). This result supports previous studies [21,50] where a combined approached outperformed a single index approach. We do however acknowledge that our study did not consider all the indices available in the literature as this was beyond the scope of the study (our aim was to only consider the most relevant indices as outline in the Methods section). Accordingly, we do not assert that a combined-indices approach will universally yield superior model performance relative to a single-index approach; however, within the context of the present study, the combined approach demonstrated improved outcomes.

By z-scoring ACT, AEI, Hf and Sm onto a common scale, the composite provides a consensus metric: it increases mainly when multiple indices jointly indicate higher “acoustic complexity” that we expect to reflect greater vocal diversity and is down-weighted when indices diverge. In this way, the composite reinforces shared soundscape trends rather than relying on a single acoustic dimension. As soundscapes are subject to stochastic variation in signal arrangement and are influenced by environmental properties and masking events [51,52], combining indices can help offset the limitations of any single metric across the dataset, resulting in more consistent predictions [17,50]. In [17], a useful parallel is provided for interpreting composite- versus single-index performance. Working with soundscape recordings from west Brisbane, including open-forest habitats, they computed 14 acoustic indices at 1-min resolution across a full 24-h record and ranked each minute using an “acoustic richness” score derived from either a single index or a weighted combination of two or more indices. They reported that index combinations provided more ecologically useful information than single metrics, improving the efficiency of bird-richness estimation (i.e., more species identified for a fixed listening effort), and they also showed that the same combinations can help detect or avoid prolonged “acoustic regimes” (e.g., cicada choruses and heavy rain) that otherwise dominate recordings [17]. Consistent with this, [50] evaluated 26 indices plus simple descriptors such as energy metrics across temperate and tropical habitat gradients and found that compound (multi-index) information was generally a stronger predictor of avian species richness than any single index, particularly in the temperate system [50]. Conversely, [53] highlight that even large multi-index models can remain context-limited: using 60 indices combined in a machine-learning framework, predictive strength differed markedly among regions (R² = 0.06 Cyprus; 0.31 China; 0.52 Australia), and no single index set transferred reliably across all areas, which also aligned with [50] report when comparing temperate versus tropical soundscapes. The computational design of Hf estimates the density of frequency occupancy, aligning with the well-defined, energy-dense bands produced by stacked callers in summer choruses of Australian open forests. However, because chorus density for a given sonotype can vary across recordings, Hf may fluctuate even when the sonotype count remains constant, helping to explain residual variance in single-index models predicting annotated sonotype counts. Additionally, Hf values decreased in highly diverse audio samples. In theory, highly diverse communities are rarely evenly composed [54], so neither Hf nor the Shannon index would approach an idealized value of 1. The predominance of choruses (energy-dense frequency bands) captured by Hf much like Shannon reflects the number of observed individuals may explains this decrease in the most diverse biophony. Sm and AEI complemented each other by capturing different scales of spectral community structure and acoustic-space use: Sm provides a finer grained assessment that is sensitive to FFT settings and dB threshold, whereas AEI quantified energy distribution across broader 1-kHz bands, offering additional perspective on frequency occupancy. Likewise, ACT provided complementary temporal information by quantifying the proportion of frames with acoustic energy above the background-noise threshold, which tends to be high in 1-minute clips dominated by continuous Orthopteran stridulation. Consistent with this interpretation, the AEI + Hf + Sm composite explained 45% of the variation in insect richness, rising to 77% after ACT was incorporated in the filtered dataset analysis.

Audio filtering altered taxon-specific prediction performance. In the raw recordings, the strongest models aligned with overall biophony, whereas in the low-cut filtered recordings the strongest fit prediction model fit insect richness. In the raw audio files, a higher background noise floor required higher Sm thresholds to avoid saturation (ceiling effects) while still computing biological signals, but these higher cut-offs likely excluded low-amplitude insect signals (see S3 and S4 Figs). Because annotation counted the full range of sonotype diversity (including diffuse “fog-like” bands and low-level click sequence visible in spectrograms), higher dB thresholds removed information that contributed to index–sonotype agreement. Thus, removing of the noisiest low-frequency band reduced the background floor, increased the effective biological signal-to-noise ratio, and allowed Sm to capture insect acoustic diversity more accurately, improving agreement between index-based estimates and annotated sonotype counts.

Support for this mechanism comes from threshold comparisons. In the raw data, the lowest usable Sm threshold that still computed biological signals (Sm dB = 5.2, based on GLM performance) explained 43% of the variation in insect richness and 38% in biophony richness. In contrast, the lowest threshold in the filtered data (Sm dB = 3.8) explained 77% of insect richness and 45% of biophony richness (S5 Table). When prediction focused on overall biophony, the best raw-data model explained 59% of richness variation (Sm dB = 6.3), while insect performance decreased to 29%. In the filtered dataset, the best biophony model explained 52% of the variation while also explaining 64% of insect richness (Sm dB = 4.3; S6 Table). Notably, these filtering and threshold related effects were evident only in the z-scored composite indices that included Sm.

We found that monitoring across the entire season and selecting recordings from consistently favourable weather conditions on the same dates at all study sites produced high-quality samples, while averaging indices yielded stronger associations with sonotype counts. A low-frequency cut-off at 300 Hz also produced a cleaner dataset for sonotype prediction models, consistent with the filtering approaches used in previous studies: 0–482 Hz [17], 0–300 Hz [50], 0–500 Hz [53].

In addition to supporting proof of concept for indices to track changes in sonotype richness, insights related to ecological patterns within the study areas can also be revealed from the index analysis. Overall, the index ordination closely matched the insect sonotype ordination (Fig 2), and dissimilarity in vocal diversity followed a comparable pattern between and within reserves for both insect sonotype counts and the z-scored equal-weight composite of indices (ACT, AEI, Hf, Sm). For insect, mean between-reserve dissimilarity (0.55) was similar to within-reserve dissimilarity (Grandchester = 0.54; Hidden Vale = 0.50), and the reserve effect was weak and non-significant (PERMANOVA p = 0.11; R² = 0.14). For the composite indices, dissimilarity was lower overall as it was calculated from recording containing other shared biophony that could have contributed to index output, but the same pattern held (between = 0.38; within Grandchester = 0.40; within Hidden Vale = 0.35; PERMANOVA p = 0.38; R² = 0.09). Dispersion did not differ detectably between reserves for either dataset (PERMDISP p = 0.28 for insects; p = 0.73 for indices), consistent with the broadly similar ellipse sizes in Fig 2. Together, these results indicate moderate species turnover (i.e., nocturnal vocal diversity) that is generally no greater across reserve boundaries than among sites within each reserve. Because invertebrates are closely associated with tree communities [55,56]), higher turnover can be expected where tree diversity and its spatial arrangement vary [57]. Consistent with this, logged Bornean forests show simplified soundscapes and homogenized vocal communities captured by Sm [58]. Orthopterans span multiple guilds across below- and above-ground microhabitats (litter, grasses, understory, and canopy) whose varying combinations across the landscape shape assemblage composition, particularly in protected forests [59,60]. An Australian open eucalypt forest with intermediate crown cover and a mosaic of litter, grasses, shrubs, and canopy is therefore likely to support a wide range of orthopteran guilds. High dissimilarity can also reflect limited sampling effort. In a study of beetle assemblages in Eucalyptus forests, sampling only a few trees rather than broader spatial coverage exaggerated estimates of dissimilarity [61], reinforcing the importance of adequate spatial replication when interpreting turnover.

In conclusion, this study highlights a case study and analysis where acoustic indices successfully reflected variation in nighttime sonotype richness. We therefore see this monitoring approach as valuable to help advance the methodological challenges often associated with surveying nighttime ecology. Although the general methodological framework applied in this study is adaptable to other environmental contexts, the performance outcomes of the indices are unlikely to be directly transferable across environments. This is because differences in vocal assemblages and associated background noise conditions are expected to influence index behaviour and resulting performance metrics. As far as assessing habitat condition, we see the use of acoustic indices not in isolation but rather as complementary to other more traditional monitoring approaches such as vegetation surveys, since fauna and flora provide mutually reinforcing perspectives on ecosystem status and considering only one risks a partial view (e.g., structurally preserved vegetation can still mask faunally depauperate habitats).

## Supporting information

S1 Fig Histogram showing the empirical distribution of full-spectrum power (0–20 kHz) among 900 candidate 1-minute audio clips, expressed in relative dB. For each clip, mean power spectral density (PSD) was estimated within consecutive 1-kHz bands, summed in linear units across the full frequency range, and then converted to relative dB to obtain the metric termed full-spectrum power.
(TIF)

S2 Fig Heatmap showing the average power spectral density (PSD; dB/Hz) across consecutive 1-kHz frequency bands from 0 to 20 kHz in a summer eucalypt open-forest. A total of 900 one-minute audio clips, collected at 10-minute intervals within a 2.5-hour sampling window, were averaged by frequency band and time point to characterize the spectral structure associated with the onset of Orthoptera activity during the reproductive season. The dataset was collected across twelve sites on January 8, 17, 21, and 26, and February 2, 2024.
(TIF)

S3 Fig Mean power spectral density (PSD) of summer nocturnal soundscapes in an Australian open-forest system, computed from 60 1-min recordings (12 sites × 5 calm, dry nights: 8, 17, 21, 26 Jan and 2 Feb 2024). For each site-night, recordings were sampled every 10 min between 19:30–22:00 h, and one file was randomly selected for analysis, totalling five 1-min audio per site, represented as autonomous recording unit (ARU). Spectra are shown for the raw recordings.
(TIF)

S4 Fig Mean power spectral density (PSD) of summer nocturnal soundscapes in an Australian open-forest system, computed from 60 1-min recordings (12 sites × 5 calm, dry nights: 8, 17, 21, 26 Jan and 2 Feb 2024). For each site-night, recordings were sampled every 10 min between 19:30–22:00 h, and one file was randomly selected for analysis. An autonomous recording unit (ARU) was deployed at each site. Spectra are shown for the recordings high-pass filtered at 300 Hz (retaining 300–20,000 Hz) to attenuate low-frequency anthropogenic noise and improve effective signal-noise-ratio (SNR).
(TIF)

S1 Table Sonotype description. Legend: Insects = predominantly crickets, grasshoppers, katydids, and locusts; Bird; Frog; Mam = mammals.
(PDF)

S2 Table Generalized linear mixed models relating clip-level annotated sonotype richness to acoustic indices in nocturnal soundscapes across 12 sites (60 one-minute files; five per site), fitted as richness ~ index + Reserve + (1|Site) for main-effect indices (ACT, AEI, Hf, Sm) and equal-weighted sums of z-scored indices (e.g., Hf + Sm, AEI + Hf + Sm, ACT + Hf + Sm, ACT + AEI + Hf + Sm). Responses include total Biophony richness, insect richness and frogs_bird_mam richness, where Biophony is the sum of annotated sonotypes from insects, frogs, birds and mammals, insect richness is the sum of annotated sonotypes, predominantly from crickets, grasshoppers, katydids and locusts, and frogs/bird/mam richness is the sum of annotated sonotypes from frogs, nocturnal birds and mammals.
(PDF)

S3 Table Generalized linear mixed models relating clip-level annotated sonotype richness to acoustic indices in nocturnal soundscapes across 12 sites (60 one-minute files; five per site), fitted as richness ~ index + Reserve + (1|Site) + (1|Nitght) for main-effect indices (ACT, AEI, Hf, Sm) and equal-weighted sums of z-scored indices (e.g., Hf + Sm, AEI + Hf + Sm, ACT + Hf + Sm, ACT + AEI + Hf + Sm). Responses include total Biophony richness, insect richness and frogs_bird_mam richness, where Biophony is the sum of annotated sonotypes from insects, frogs, birds and mammals, insect richness is the sum of annotated sonotypes, predominantly from crickets, grasshoppers, katydids and locusts, and frogs/bird/mam richness is the sum of annotated sonotypes from frogs, nocturnal birds and mammals.
(PDF)

S4 Table Generalized Linear Models of annotated sonotype richness versus acoustic indices calculated from nocturnal soundscapes across 12 sites, using predictor variables for main-effect indices (ACT, AEI, Hf, Sm) and equal-weighted sums of z-scored indices (e.g., Hf + Sm, AEI + Hf + Sm, ACT + Hf + Sm, ACT + AEI + Hf + Sm). Responses include site-level mean richness for (i) Biophony, defined as the mean number of annotated sonotypes pooled across insects, frogs, birds, and mammals; (ii) Insects, defined as the mean number of annotated sonotypes from crickets, grasshoppers, katydids, and locusts; and (iii) frogs/bird/mam, defined as the mean number of annotated sonotypes from frogs, nocturnal birds, and mammals.
(PDF)

S5 Table Generalized Linear Models of annotated sonotype richness versus acoustic indices calculated from nocturnal soundscapes across 12 sites, using predictor variables for main-effect indices (ACT, AEI, Hf, Sm) and equal-weighted sums of z-scored indices (e.g., Hf + Sm, AEI + Hf + Sm, ACT + Hf + Sm, ACT + AEI + Hf + Sm). Responses include site-level accumulated richness for (i) Biophony, defined as the total number of unique annotated sonotypes pooled across insects, frogs, birds, and mammals; (ii) Insects, defined as the total number of unique annotated sonotypes, predominantly from crickets, grasshoppers, katydids, and locusts; and (iii) frogs/bird/mam, defined as the total number of unique annotated sonotypes from frogs, nocturnal birds, and mammals. Dataset filtered refers to recordings filtered to remove low-frequency noise (300 Hz–20 kHz); dataset raw_1 min refers to unfiltered recordings spanning 0–20 kHz. dB denotes the decibel threshold used for the soundscape saturation index (Sm), and Disp denotes dispersion after fitting the selected model family.
(PDF)

S6 Table Generalized Linear Models of annotated sonotype richness versus acoustic indices calculated from nocturnal soundscapes across 12 sites, using predictor variables for main-effect indices (ACT, AEI, Hf, Sm) and equal-weighted sum of z-scored indices (e.g., Hf + Sm, AEI + Hf + Sm, ACT + Hf + Sm, ACT + AEI + Hf + Sm). Responses include site-level accumulated richness for (i) Biophony, defined as the total number of unique annotated sonotypes pooled across insects, frogs, birds, and mammals; (ii) Insects, defined as the total number of unique annotated sonotypes, predominantly from crickets, grasshoppers, katydids, and locusts; and (iii) frogs/bird/mam, defined as the total number of unique annotated sonotypes from frogs, nocturnal birds, and mammals. Dataset filtered refers to recordings filtered to remove low-frequency noise (300 Hz–20 kHz); dataset raw_1 min refers to unfiltered recordings spanning 0–20 kHz. dB denotes the decibel threshold used for the soundscape saturation index (Sm), and Disp denotes dispersion after fitting the selected model family.
(PDF)
