# Supplementary material for: Exploring nocturnal soundscapes in an Australian open forest system using acoustic indices
Source: PLoS One. 2026 May 15;21(5):e0348624. doi: 10.1371/journal.pone.0348624 (PMC13178895; doi:10.1371/journal.pone.0348624)
Supplement: S1 Table — Legend: Insects = predominantly crickets, grasshoppers, katydids, and locusts; Bird; Frog; Mam = mammals. (PDF) [file pone.0348624.s005.pdf]

Sonotypes	Frequency range (Hz)
Insect1	1400 - 3800
Insect2	1800 - 2200
Insect3	1800 - 2500
Insect4	2000 - 2400
Insect5	2400 - 3300
Insect6	3200 - 3800
Insect7	3400 - 3800
Insect8	3800 - 4400
Insect9	3500 - 4000
Insect10	3500 - 4800
Insect11	3500 - 5500
Insect12	4000 - 4400
Insect13	4300 - 4500
Insect14	4800 - 5800
Insect15	4800 - 5200
Insect16	4800 - 5200
Insect17	5000 - 5500
Insect18	5200- 6200
Insect19	5500 - 5900
Insect20	5500 - 6500
Insect21	6000 - 10000
Insect22	6500 - 7000
Insect23	6000 - 14000
Insect24	6500 - 9000
Insect25	7000 - 15000
Insect26	7500 - 8500
Insect27	8000 - 8500
Insect28	8000 - 15000
Insect29	9000 - 15000
Insect30	9000 - 15000
Insect31	10000 - 16000
Insect32	10000 - 19000
Insect33	10000 - 20000
Insect34	10500 - 12000
Insect35	12000 - 14000
Insect36	12000 - 20000
Insect37	12000 - 20000

Sonotypes	Frequency range (Hz)
Bird1	300 - 400
Bird2	500 - 800
Bird3	500 - 1400
Bird4	900 - 3000
Bird5	1000 - 3500
Bird6	1200 - 3500
Bird7	1100 - 1500
Bird8	1200 - 4000
Bird9	1400 - 3600
Bird10	2000- 4000
Frog1	400 - 2000
Frog2	400 - 1200
Frog3	500 - 1000
Frog4	500 - 2500
Frog5	1200 - 3500
Frog6	2000 - 3000
Mam1	300 - 600
Mam2	300 - 1200
Mam3	300 - 3500
Mam4	300 - 1500
Mam5	300 - 3000
Mam6	300 - 1000
Mam7	400-800
Mam8	500 - 2000
Mam9	1000 - 2000
Mam10	2000 - 4000
Mam11	10000 - 19000
