# Supplementary material for: Exploring nocturnal soundscapes in an Australian open forest system using acoustic indices
Source: PLoS One. 2026 May 15;21(5):e0348624. doi: 10.1371/journal.pone.0348624 (PMC13178895; doi:10.1371/journal.pone.0348624)
Supplement: S2 Table — Responses include total Biophony richness, insect richness and frogs_bird_mam richness, where Biophony is the sum of annotated sonotypes from insects, frogs, birds and mammals, insect richness is the sum of annotated sonotypes, predominantly from crickets, grasshoppers, katydids and locusts, and frogs/bird/mam richness is the sum of annotated sonotypes from frogs, nocturnal birds and mammals. (PDF) [file pone.0348624.s006.pdf]

Dataset	Response	Index	Family	Disp	p-value	R^2	AICc
Filtered	Frogs/Birds/Mammals	z-scored (ACT, AEI, Hf, Sm)	COMPOIS	0.93	0.03	0.12	214
Filtered	Frogs/Birds/Mammals	ACT	COMPOIS	0.93	0.08	0.11	216
Filtered	Frogs/Birds/Mammals	AEI	COMPOIS	0.93	0.30	0.07	218
Filtered	Frogs/Birds/Mammals	z-scored (ACT, Hf, Sm)	COMPOIS	0.91	0.42	0.08	218
Filtered	Frogs/Birds/Mammals	z-scored (AEI, Hf, Sm)	COMPOIS	0.91	0.70	0.07	219
Filtered	Frogs/Birds/Mammals	z-scored (Hf, Sm)	COMPOIS	0.92	0.74	0.07	219
Filtered	Frogs/Birds/Mammals	Hf	COMPOIS	0.92	0.76	0.06	219
Filtered	Frogs/Birds/Mammals	Sm	COMPOIS	0.91	0.79	0.07	219
Filtered	Insects	Hf	COMPOIS	1.03	0.35	0.25	247
Filtered	Insects	AEI	COMPOIS	1.02	0.42	0.24	247
Filtered	Insects	z-scored (ACT, Hf, Sm)	COMPOIS	1.03	0.53	0.25	248
Filtered	Insects	Sm	COMPOIS	1.04	0.58	0.22	248
Filtered	Insects	ACT	COMPOIS	1.04	0.68	0.23	248
Filtered	Insects	z-scored (AEI, Hf, Sm)	COMPOIS	1.03	0.69	0.21	248
Filtered	Insects	z-scored (Hf, Sm)	COMPOIS	1.02	0.80	0.23	248
Filtered	Insects	z-scored (ACT, AEI, Hf, Sm)	COMPOIS	1.02	0.97	0.22	248
Filtered	Biophony	ACT	COMPOIS	0.99	0.13	0.04	271
Filtered	Biophony	z-scored (ACT, AEI, Hf, Sm)	COMPOIS	0.99	0.33	0.07	272
Filtered	Biophony	Sm	COMPOIS	0.98	0.40	0.02	272
Filtered	Biophony	z-scored (ACT, Hf, Sm)	COMPOIS	0.99	0.47	0.07	272
Filtered	Biophony	z-scored (AEI, Hf, Sm)	COMPOIS	1	0.67	0.01	273
Filtered	Biophony	Hf	COMPOIS	1	0.76	0.01	273
Filtered	Biophony	z-scored (Hf, Sm)	COMPOIS	0.99	0.77	0.01	273
Filtered	Biophony	AEI	COMPOIS	1	0.97	0.01	273
Raw_1min	Frogs/Birds/Mammals	ACT	COMPOIS	0.92	0.10	0.1	216
Raw_1min	Frogs/Birds/Mammals	z-scored (ACT, AEI, Hf, Sm)	COMPOIS	0.91	0.13	0.1	216
Raw_1min	Frogs/Birds/Mammals	AEI	COMPOIS	0.91	0.27	0.08	217
Raw_1min	Frogs/Birds/Mammals	Sm	COMPOIS	0.91	0.41	0.07	218
Raw_1min	Frogs/Birds/Mammals	Hf, Sm	COMPOIS	0.92	0.53	0.07	218
Raw_1min	Frogs/Birds/Mammals	Hf	COMPOIS	0.91	0.70	0.06	219
Raw_1min	Frogs/Birds/Mammals	z-scored (ACT, Hf, Sm)	COMPOIS	0.91	0.82	0.07	219
Raw_1min	Frogs/Birds/Mammals	z-scored (AEI, Hf, Sm)	COMPOIS	0.92	0.95	0.06	219
Raw_1min	Insects	z-scored (ACT, Hf, Sm)	COMPOIS	1.03	0.13	0.28	246
Raw_1min	Insects	z-scored (ACT, AEI, Hf, Sm)	COMPOIS	1.02	0.19	0.29	246
Raw_1min	Insects	AEI	COMPOIS	1.02	0.26	0.24	247
Raw_1min	Insects	z-scored (Hf, Sm)	COMPOIS	1.02	0.27	0.26	247
Raw_1min	Insects	Sm	COMPOIS	1.01	0.28	0.25	247
Raw_1min	Insects	Hf	COMPOIS	1.04	0.31	0.26	247
Raw_1min	Insects	z-scored (AEI, Hf, Sm)	COMPOIS	1.04	0.39	0.26	247
Raw_1min	Insects	ACT	COMPOIS	1.03	0.78	0.23	248
Raw_1min	Biophony	z-scored (ACT, AEI, Hf, Sm)	COMPOIS	1.01	0.10	0.13	270
Raw_1min	Biophony	ACT	COMPOIS	0.99	0.20	0.03	271
Raw_1min	Biophony	z-scored (ACT, Hf, Sm)	COMPOIS	1	0.26	0.07	271
Raw_1min	Biophony	Hf	COMPOIS	0.99	0.74	0.01	273
Raw_1min	Biophony	z-scored (Hf, Sm)	COMPOIS	0.98	0.77	0.01	273
Raw_1min	Biophony	z-scored (AEI, Hf, Sm)	COMPOIS	1.01	0.78	0.01	273
Raw_1min	Biophony	AEI	COMPOIS	1.01	0.83	0.01	273
Raw_1min	Biophony	Sm	COMPOIS	0.99	0.83	0.01	273
