# Supplementary material for: Exploring nocturnal soundscapes in an Australian open forest system using acoustic indices
Source: PLoS One. 2026 May 15;21(5):e0348624. doi: 10.1371/journal.pone.0348624 (PMC13178895; doi:10.1371/journal.pone.0348624)
Supplement: S3 Table — Responses include total Biophony richness, insect richness and frogs_bird_mam richness, where Biophony is the sum of annotated sonotypes from insects, frogs, birds and mammals, insect richness is the sum of annotated sonotypes, predominantly from crickets, grasshoppers, katydids and locusts, and frogs/bird/mam richness is the sum of annotated sonotypes from frogs, nocturnal birds and mammals. (PDF) [file pone.0348624.s007.pdf]

Dataset	Response	Index	Family	Disp	p-value	R^2	AICc
Filtered	Frogs/Birds/Mammals	ACT	COMPOIS	0.92	0.18	0.32	214
Filtered	Frogs/Birds/Mammals	z-scored (ACT, AEI, Hf, Sm)	COMPOIS	0.93	0.25	0.3	214
Filtered	Frogs/Birds/Mammals	AEI	COMPOIS	0.9	0.26	0.32	215
Filtered	Frogs/Birds/Mammals	Hf	COMPOIS	0.89	0.42	0.32	215
Filtered	Frogs/Birds/Mammals	z-scored (Hf, Sm)	COMPOIS	0.92	0.46	0.32	215
Filtered	Frogs/Birds/Mammals	Sm	COMPOIS	0.89	0.60	0.32	216
Filtered	Frogs/Birds/Mammals	z-scored (AEI, Hf, Sm)	COMPOIS	0.91	0.87	0.31	216
Filtered	Frogs/Birds/Mammals	z-scored (ACT, Hf, Sm)	COMPOIS	0.92	0.97	0.31	216
Filtered	Insects	Hf	COMPOIS	1.03	0.35	0.25	250
Filtered	Insects	AEI	COMPOIS	1.02	0.43	0.24	250
Filtered	Insects	z-scored (ACT, Hf, Sm)	COMPOIS	1.03	0.53	0.25	250
Filtered	Insects	Sm	COMPOIS	1.02	0.60	0.23	250
Filtered	Insects	z-scored (AEI, Hf, Sm)	COMPOIS	1.05	0.69	0.23	250
Filtered	Insects	ACT	COMPOIS	1.02	0.69	0.24	250
Filtered	Insects	Hf, Sm	COMPOIS	1.05	0.82	0.25	251
Filtered	Insects	z-scored (ACT, AEI, Hf, Sm)	COMPOIS	1.01	0.96	0.24	251
Filtered	Biophony	ACT	COMPOIS	1.01	0.21	0.16	272
Filtered	Biophony	Sm	COMPOIS	1.01	0.37	0.16	273
Filtered	Biophony	z-scored (AEI, Hf, Sm)	COMPOIS	1	0.42	0.16	273
Filtered	Biophony	Hf, Sm	COMPOIS	1.02	0.55	0.16	273
Filtered	Biophony	z-scored (ACT, AEI, Hf, Sm)	COMPOIS	1.01	0.68	0.2	273
Filtered	Biophony	z-scored (ACT, Hf, Sm)	COMPOIS	1.01	0.80	0.21	273
Filtered	Biophony	AEI	COMPOIS	1	0.90	0.19	273
Filtered	Biophony	Hf	COMPOIS	1.02	0.91	0.19	273
Raw_1min	Frogs/Birds/Mammals	ACT	COMPOIS	0.9	0.18	0.32	214
Raw_1min	Frogs/Birds/Mammals	AEI	COMPOIS	0.94	0.21	0.33	214
Raw_1min	Frogs/Birds/Mammals	z-scored (ACT, AEI, Hf, Sm)	COMPOIS	0.92	0.23	0.32	214
Raw_1min	Frogs/Birds/Mammals	Hf	COMPOIS	0.89	0.37	0.33	215
Raw_1min	Frogs/Birds/Mammals	z-scored (Hf, Sm)	COMPOIS	0.88	0.45	0.32	215
Raw_1min	Frogs/Birds/Mammals	Sm	COMPOIS	0.91	0.56	0.31	215
Raw_1min	Frogs/Birds/Mammals	z-scored (AEI, Hf, Sm)	COMPOIS	0.92	0.89	0.31	216
Raw_1min	Frogs/Birds/Mammals	z-scored (ACT, Hf, Sm)	COMPOIS	0.91	0.99	0.31	216
Raw_1min	Insects	z-scored (ACT, Hf, Sm)	COMPOIS	1.04	0.13	0.28	248
Raw_1min	Insects	z-scored (ACT, AEI, Hf, Sm)	COMPOIS	1.03	0.19	0.29	249
Raw_1min	Insects	AEI	COMPOIS	1.04	0.26	0.24	249
Raw_1min	Insects	z-scored (Hf, Sm)	COMPOIS	1.04	0.27	0.26	249
Raw_1min	Insects	Sm	COMPOIS	1.02	0.28	0.25	249
Raw_1min	Insects	Hf	COMPOIS	1.02	0.31	0.26	250
Raw_1min	Insects	z-scored (AEI, Hf, Sm)	COMPOIS	1.03	0.39	0.26	250
Raw_1min	Insects	ACT	COMPOIS	1.02	0.79	0.24	251
Raw_1min	Biophony	z-scored (ACT, AEI, Hf, Sm)	COMPOIS	1	0.22	0.23	272
Raw_1min	Biophony	ACT	COMPOIS	1.01	0.30	0.18	272
Raw_1min	Biophony	z-scored (ACT, Hf, Sm	COMPOIS	1.02	0.39	0.22	273
Raw_1min	Biophony	Sm	COMPOIS	1	0.78	0.21	273
Raw_1min	Biophony	z-scored (AEI, Hf, Sm)	COMPOIS	1	0.91	0.2	273
Raw_1min	Biophony	z-scored (Hf, Sm)	COMPOIS	1.02	0.92	0.2	273
Raw_1min	Biophony	Hf	COMPOIS	1	0.92	0.19	273
Raw_1min	Biophony	AEI	COMPOIS	1.01	0.95	0.19	273
