# Supplementary material for: Exploring nocturnal soundscapes in an Australian open forest system using acoustic indices
Source: PLoS One. 2026 May 15;21(5):e0348624. doi: 10.1371/journal.pone.0348624 (PMC13178895; doi:10.1371/journal.pone.0348624)
Supplement: S4 Table — Responses include site-level mean richness for (i) Biophony, defined as the mean number of annotated sonotypes pooled across insects, frogs, birds, and mammals; (ii) Insects, defined as the mean number of annotated sonotypes from crickets, grasshoppers, katydids, and locusts; and (iii) frogs/bird/mam, defined as the mean number of annotated sonotypes from frogs, nocturnal birds, and mammals. (PDF) [file pone.0348624.s008.pdf]

Dataset	Response	Index	Family	Disp	p-value	R^2	AICc
Filtered	Frogs/Birds/Mammals	z-scored (ACT, AEI, Hf, Sm)	COMPOIS	0.98	0.093	0.44	36
Filtered	Frogs/Birds/Mammals	ACT	COMPOIS	1.00	0.492	0.32	38
Filtered	Frogs/Birds/Mammals	z-scored (ACT, Hf, Sm)	COMPOIS	1.01	0.465	0.32	38
Filtered	Frogs/Birds/Mammals	z-scored (AEI, Hf, Sm)	COMPOIS	0.95	0.535	0.33	38
Filtered	Frogs/Birds/Mammals	Hf	COMPOIS	1.01	0.465	0.31	38
Filtered	Frogs/Birds/Mammals	z-scored (Hf, Sm)	COMPOIS	1.02	0.406	0.32	38
Filtered	Frogs/Birds/Mammals	Sm	COMPOIS	1.00	0.426	0.33	38
Filtered	Insect	z-scored (ACT, AEI, Hf, Sm)	COMPOIS	1.11	0.003	0.43	41
Filtered	Biophony	z-scored (Hf, Sm)	COMPOIS	1.12	0.027	0.3	42
Filtered	Biophony	Sm	COMPOIS	1.14	0.028	0.29	42
Filtered	Biophony	z-scored (ACT, Hf, Sm)	COMPOIS	1.11	0.037	0.27	43
Filtered	Biophony	AEI	COMPOIS	1.11	0.072	0.21	43
Filtered	Frogs/Birds/Mammals	AEI	POIS	0.14	0.304	0.6	43
Filtered	Biophony	z-scored (AEI, Hf, Sm)	COMPOIS	1.12	0.127	0.17	44
Filtered	Biophony	Hf	COMPOIS	1.11	0.089	0.2	44
Filtered	Insect	z-scored (AEI, Hf, Sm)	COMPOIS	1.15	0.087	0.21	44
Filtered	Biophony	ACT	COMPOIS	1.12	0.189	0.13	45
Filtered	Insect	z-scored (ACT, Hf, Sm)	COMPOIS	1.12	0.104	0.2	45
Filtered	Biophony	z-scored (ACT, AEI, Hf, Sm)	COMPOIS	1.08	0.397	0.06	46
Filtered	Insect	Hf	COMPOIS	1.12	0.286	0.11	46
Filtered	Insect	z-scored (Hf, Sm)	COMPOIS	1.11	0.268	0.11	46
Filtered	Insect	Sm	COMPOIS	1.11	0.348	0.09	46
Filtered	Insect	ACT	COMPOIS	1.11	0.973	0.03	47
Filtered	Insect	AEI	COMPOIS	1.11	0.988	0.03	47
Raw_1min	Frogs/Birds/Mammals	AEI	COMPOIS	1.41	0.005	0.57	30
Raw_1min	Frogs/Birds/Mammals	Sm	COMPOIS	1.08	0.164	0.36	36
Raw_1min	Frogs/Birds/Mammals	z-scored (ACT, Hf, Sm)	COMPOIS	1.02	0.303	0.34	37
Raw_1min	Frogs/Birds/Mammals	Hf	COMPOIS	1.03	0.351	0.33	37
Raw_1min	Frogs/Birds/Mammals	z-scored (Hf, Sm)	COMPOIS	1.04	0.246	0.34	37
Raw_1min	Frogs/Birds/Mammals	ACT	COMPOIS	1.03	0.567	0.32	38
Raw_1min	Frogs/Birds/Mammals	z-scored (ACT, AEI, Hf, Sm)	COMPOIS	0.94	0.588	0.33	38
Raw_1min	Frogs/Birds/Mammals	z-scored (AEI, Hf, Sm)	COMPOIS	0.95	0.936	0.3	38
Raw_1min	Biophony	z-scored (AEI, Hf, Sm)	COMPOIS	1.10	0.074	0.22	43
Raw_1min	Biophony	Hf	COMPOIS	1.09	0.074	0.22	43
Raw_1min	Biophony	z-scored (Hf, Sm)	COMPOIS	1.10	0.072	0.22	43
Raw_1min	Biophony	z-scored (ACT, Hf, Sm)	COMPOIS	1.08	0.145	0.15	44
Raw_1min	Biophony	Sm	COMPOIS	1.11	0.079	0.21	44
Raw_1min	Insect	z-scored (ACT, AEI, Hf, Sm)	COMPOIS	1.11	0.052	0.26	44
Raw_1min	Biophony	ACT	COMPOIS	1.09	0.370	0.07	45
Raw_1min	Biophony	z-scored (ACT, AEI, Hf, Sm)	COMPOIS	1.13	0.265	0.1	45
Raw_1min	Biophony	AEI	COMPOIS	1.13	0.251	0.1	45
Raw_1min	Insect	z-scored (AEI, Hf, Sm)	COMPOIS	1.14	0.117	0.19	45
Raw_1min	Insect	z-scored (ACT, Hf, Sm)	COMPOIS	1.13	0.376	0.08	46
Raw_1min	Insect	Hf	COMPOIS	1.11	0.306	0.1	46
Raw_1min	Insect	ACT	COMPOIS	1.09	0.873	0.03	47
Raw_1min	Insect	AEI	COMPOIS	1.13	0.695	0.04	47
Raw_1min	Insect	z-scored (Hf, Sm)	COMPOIS	1.18	0.478	0.06	47
Raw_1min	Insect	Sm	COMPOIS	1.13	0.691	0.04	47
