# Supplementary material for: Exploring nocturnal soundscapes in an Australian open forest system using acoustic indices
Source: PLoS One. 2026 May 15;21(5):e0348624. doi: 10.1371/journal.pone.0348624 (PMC13178895; doi:10.1371/journal.pone.0348624)
Supplement: S6 Table — Responses include site-level accumulated richness for (i) Biophony, defined as the total number of unique annotated sonotypes pooled across insects, frogs, birds, and mammals; (ii) Insects, defined as the total number of unique annotated sonotypes, predominantly from crickets, grasshoppers, katydids, and locusts; and (iii) frogs/bird/mam, defined as the total number of unique annotated sonotypes from frogs, nocturnal birds, and mammals. Dataset filtered refers to recordings filtered to remove low-frequency noise (300 Hz–20 kHz); dataset raw_1 min refers to unfiltered recordings spanning 0–20 kHz. dB denotes the decibel threshold used for the soundscape saturation index (Sm), and Disp denotes dispersion after fitting the selected model family. (PDF) [file pone.0348624.s010.pdf]

Dataset	dB	Response	Index	Family	Disp	p-value	R^2	AICc
Filtered	4.3	Insects	z-scored (ACT, AEI, Hf,	COMPOIS	1.1	8.90E-06	0.64	59
Filtered	4.3	Biophony	z-scored (AEI, Hf, Sm)	COMPOIS	1.1	0.00049	0.52	57
Filtered	4.3	Insects	z-scored (AEI, Hf, Sm)	COMPOIS	1.13	0.00071	0.51	63
Filtered	4.3	Biophony	z-scored (ACT, AEI, Hf,	COMPOIS	1.07	0.00268	0.45	59
Filtered	4.3	Frogs/Birds/Mammals	AEI	COMPOIS	1.11	0.02032	0.44	55
Filtered	4.3	Biophony	Hf	COMPOIS	1.1	0.01103	0.37	60
Filtered	4.3	Biophony	z-scored (ACT, Hf, Sm)	COMPOIS	1.1	0.01492	0.35	60
Filtered	4.3	Biophony	z-scored (Hf, Sm)	COMPOIS	1.11	0.01496	0.35	60
Filtered	4.3	Frogs/Birds/Mammals	z-scored (ACT, AEI, Hf,	COMPOIS	1.08	0.09299	0.33	57
Filtered	4.3	Frogs/Birds/Mammals	Hf	COMPOIS	1.06	0.32166	0.26	59
Filtered	4.3	Biophony	Sm	COMPOIS	1.13	0.05259	0.25	62
Filtered	4.3	Frogs/Birds/Mammals	ACT	COMPOIS	1.01	0.46112	0.24	59
Filtered	4.3	Frogs/Birds/Mammals	z-scored (AEI, Hf, Sm)	COMPOIS	1.08	0.47425	0.23	59
Filtered	4.3	Frogs/Birds/Mammals	z-scored (Hf, Sm)	COMPOIS	1.03	0.56177	0.23	59
Filtered	4.3	Frogs/Birds/Mammals	z-scored (ACT, Hf, Sm)	COMPOIS	1.05	0.74421	0.22	60
Filtered	4.3	Frogs/Birds/Mammals	Sm	COMPOIS	1.03	0.87812	0.21	60
Filtered	4.3	Insects	z-scored (ACT, Hf, Sm)	COMPOIS	1.12	0.13624	0.18	69
Filtered	4.3	Insects	z-scored (Hf, Sm)	COMPOIS	1.12	0.19447	0.15	69
Filtered	4.3	Insects	Sm	COMPOIS	1.1	0.1792	0.15	69
Filtered	4.3	Insects	Hf	COMPOIS	1.14	0.28889	0.11	70
Filtered	4.3	Biophony	ACT	COMPOIS	1.08	0.32662	0.1	64
Filtered	4.3	Insects	AEI	COMPOIS	1.14	0.4822	0.07	70
Filtered	4.3	Biophony	AEI	COMPOIS	1.14	0.51119	0.06	65
Filtered	4.3	Insects	ACT	COMPOIS	1.11	0.77225	0.04	71
Raw_1min	6.3	Biophony	z-scored (AEI, Hf, Sm)	COMPOIS	1.08	6.76E-05	0.59	55
Raw_1min	6.3	Frogs/Birds/Mammals	AEI	COMPOIS	1.05	0.01978	0.44	55
Raw_1min	6.3	Frogs/Birds/Mammals	Sm	COMPOIS	1.04	0.09503	0.34	57
Raw_1min	6.3	Frogs/Birds/Mammals	z-scored (Hf, Sm)	COMPOIS	1.07	0.15584	0.31	58
Raw_1min	6.3	Frogs/Birds/Mammals	z-scored (ACT, Hf, Sm)	COMPOIS	1.09	0.17243	0.3	58
Raw_1min	6.3	Biophony	z-scored (ACT, AEI, Hf,	COMPOIS	1.08	0.00257	0.45	59
Raw_1min	6.3	Frogs/Birds/Mammals	Hf	COMPOIS	1.07	0.25129	0.28	59
Raw_1min	6.3	Frogs/Birds/Mammals	z-scored (AEI, Hf, Sm)	COMPOIS	1.01	0.63647	0.22	60
Raw_1min	6.3	Frogs/Birds/Mammals	ACT	COMPOIS	1.02	0.64367	0.22	60
Raw_1min	6.3	Frogs/Birds/Mammals	z-scored (ACT, AEI, Hf,	COMPOIS	1	0.86612	0.21	60
Raw_1min	6.3	Biophony	Hf	COMPOIS	1.08	0.01674	0.34	61
Raw_1min	6.3	Biophony	z-scored (Hf, Sm)	COMPOIS	1.14	0.0326	0.3	61
Raw_1min	6.3	Biophony	z-scored (ACT, Hf, Sm)	COMPOIS	1.13	0.06224	0.24	62
Raw_1min	6.3	Biophony	Sm	COMPOIS	1.13	0.06551	0.24	62
Raw_1min	6.3	Biophony	ACT	COMPOIS	1.09	0.42553	0.07	65
Raw_1min	6.3	Biophony	AEI	COMPOIS	1.1	0.78422	0.03	65
Raw_1min	6.3	Insects	z-scored (AEI, Hf, Sm)	COMPOIS	1.14	0.03705	0.29	67
Raw_1min	6.3	Insects	z-scored (ACT, AEI, Hf,	COMPOIS	1.08	0.05224	0.27	68
Raw_1min	6.3	Insects	AEI	COMPOIS	1.13	0.30855	0.11	70
Raw_1min	6.3	Insects	Hf	COMPOIS	1.1	0.37199	0.09	70
Raw_1min	6.3	Insects	Hf, Sm	COMPOIS	1.14	0.54133	0.06	71
Raw_1min	6.3	Insects	z-scored (ACT, Hf, Sm)	COMPOIS	1.09	0.61165	0.05	71
Raw_1min	6.3	Insects	Sm	COMPOIS	1.11	0.7437	0.04	71
Raw_1min	6.3	Insects	ACT	COMPOIS	1.12	0.74384	0.04	71
